# Disparities and factors affecting hypertension diagnosis from qualified doctors in Bangladesh and its impact on receiving hypertension control advice: Analysis of demographic & health survey 2017–18

**DOI:** 10.1371/journal.pgph.0003496

**Published:** 2024-07-23

**Authors:** Gulam Muhammed Al Kibria, Md Shajedur Rahman Shawon, Mohammad Rashidul Hashan, Maryam Hameed Khan, Dustin G. Gibson

**Affiliations:** 1 Department of International Health, Johns Hopkins University Bloomberg School of Public Health, Baltimore, Maryland, United States of America; 2 Centre for Big Data Research in Health, UNSW Sydney, Sydney, Australia; 3 Bangladesh Civil Service, Ministry of Health and Family Welfare, Government of Bangladesh, Dhaka, Bangladesh; Bangladesh University of Health Sciences, BANGLADESH

## Abstract

The burden of hypertension is increasing in many low- and middle-income countries, including Bangladesh, and a large proportion of Bangladeshi people seek healthcare from unqualified medical practitioners, such as paramedics, village doctors, and drug store salesmen; however, there has been limited investigation regarding diagnosis and care provided by qualified doctors. This study investigated the factors associated with hypertension diagnosis by qualified doctors (i.e., registered medically trained doctors or medical doctors with at least an MBBS degree) and how this diagnosis is related to hypertension-controlling advice and treatment among Bangladeshi adults. This cross-sectional study used data from Bangladesh Demographic and Health Survey 2017–18. After describing sample characteristics, we conducted simple and multivariable logistic regression analyses to investigate the associated factors and associations. Among 1710 participants (68.3% females, mean age: 50.1 (standard error: 0.43) years) with self-reported hypertension diagnosis, about 54.9% (95% confidence interval (CI): 51.8–58.0) had a diagnosis by qualified doctors. The following variables had significant associations with hypertension diagnoses from qualified doctors: 40-54- or 55-year-olds/above (ref: 18-29-year-olds), overweight/obesity (ref: not overweight/obese), college/above education (ref: no formal education), richest wealth quintile (ref: poorest), urban residence (ref: rural), and residence in Chittagong, Barisal, and Sylhet divisions (ref: Dhaka division). Lastly, compared to people who had not been diagnosed by qualified doctors, those with the diagnosis from qualified doctors had higher odds of receiving any hypertension-controlling advice and treatment, including drugs (1.73 (95% CI: 1.27–2.36), salt intake reduction (AOR: 2.36, 95% CI: 1.80–3.10), weight reduction (AOR: 2.58, 95% CI: 1.97–3.37), smoking cessation (AOR: 2.22, 95% CI: 1.66–2.96),), and exercise promotion (AOR: 2.34, 95% CI: 1.77–3.09). This study showed significant socioeconomic and rural-urban disparities regarding hypertension diagnosis from qualified doctors. Diagnosis by qualified doctors was also positively associated with receiving hypertension-controlling advice and treatment. Reducing these inequalities would be crucial to reducing the country’s hypertension burden.

## Background

Hypertension is one of the major risk factors for cardiovascular, cerebrovascular, and nephrotic diseases. It is a significant contributor to deaths and disabilities among adults globally [1, 2]. Recent studies indicate a substantial surge in the prevalence of hypertension in low- and middle-income countries (LMICs) [2]. Several common behavioral risk factors, such as lack of physical activity, unhealthy dietary patterns, excessive alcohol consumption, and tobacco use, are primarily responsible for this rising trend [3]. The burden of hypertension is also increasing in Bangladesh [4, 5]. According to the latest data from the Bangladesh Demographic and Health Survey (BDHS) 2017–18, a nationally representative survey, approximately 45% of women and 34% of men aged 35 and above may have hypertension. An additional 30% of people may be prehypertensive and have a higher risk of developing hypertension compared to individuals with blood pressure levels within the normal range [6]. The prevalence of hypertension represents a notable increase from the 2011 estimates, which reported prevalence rates of 32% for women and 20% for men in the same age group [7]. Apart from the previously mentioned behavioral risk factors, factors such as demographic transition, a growing aging population, and shifts in socioeconomic status (SES) likely contribute to this heightened prevalence in Bangladesh [2, 5, 8, 9].

In addition to curbing the rising prevalence of hypertension, to reduce the associated complications, it is crucial to initiate measures as early as possible. After a diagnosis of hypertension, a qualified medical doctor may recommend a combination of pharmacologic measures (e.g., antihypertensive medication) and behavioral interventions (e.g., weight reduction, dietary changes, and smoking cessation) to control it [10, 11]. As people with hypertension also have a higher risk of other noncommunicable diseases (NCDs) (e.g., diabetes and dyslipidemia), doctors may screen people with hypertension for those conditions [11].

An extensive body of literature previously reported disparities in care related to various health conditions in Bangladesh, especially maternal and child health conditions. For instance, multiple studies reported that women with higher SES (e.g., high education or wealth) are more likely to receive healthcare like antenatal care, skilled birth attendance, facility delivery, and postnatal care compared to women with lower SES (e.g., low education or wealth) [6, 12–14]. Furthermore, studies reported that rural women are less likely to receive healthcare than their urban counterparts [12, 15]. Studies that investigated the determinants of ’awareness’ of hypertension and diabetes reported similar socioeconomic and rural-urban disparities. These disparities result from healthcare services’ affordability, access, and availability [16–19].

Many Bangladeshi people seek healthcare from unqualified medical practitioners, such as paramedics, village doctors, and drug store salesmen [6, 20]. The quality of treatment, care, or advice these unqualified practitioners provide may vary compared to that of qualified doctors due to the lack of essential training and knowledge [6, 7, 20]. Overall, studies that looked into hypertension diagnosis or treatment trends reported that the proportion of people with treatment/care has increased [21, 22]. On the other hand, studies that were conducted in a particular area of Bangladesh reported that the availability of care was inadequate [23–25]. While there have been studies on the distribution and determinants of maternal/child healthcare or prevalence, control, treatment, and awareness related to hypertension, there has been limited exploration of the quality of hypertension care in Bangladesh, especially nationally representative studies. To address these gaps in knowledge, we utilized a nationally representative sample of Bangladeshi adults to estimate the prevalence and determinants of receiving a hypertension diagnosis from qualified medical doctors (i.e., registered medically trained doctors or medical doctors with at least an MBBS degree). Additionally, we investigated the association between receiving hypertension-controlling advice/treatment (i.e., medication, weight loss, salt reduction, smoking cessation, and exercise promotion) and receiving care from qualified medical doctors.

## Methods

### Study design/setting

This cross-sectional study utilized data from the BDHS 2017–18, a nationally representative household survey in Bangladesh. This was the eighth DHS in the country. Data collection took place between October 2017 and March 2018. We conducted the data analysis in November 2023 [6].

One of the primary objectives of BDHS 2017–18 was to estimate the prevalence of hypertension and diabetes among men and women in Bangladesh. The sampling process involved a stratified, two-stage selection of households. A list of enumeration areas (EAs) was derived from the 2011 Population and Housing Census of Bangladesh. EA was the primary sampling unit and was selected based on the probability proportionate to its size. The number of EAs from rural and urban regions was 425 and 250, respectively. The list of households within each EA was prepared. Approximately 30 households were randomly chosen from this list, resulting in a total of 20,250 households. One-fourth of these households were randomly selected to have blood pressure measurements. People who were at least 18 years old were eligible to participate. Detailed information on the survey design, methods, sample size calculation, questionnaires, and other relevant statistics can be accessed online [6].

### Outcomes

#### Diagnosis by qualified doctors

Participants were asked two questions related to their hypertension diagnosis: *(1) "Have you ever been told by a doctor or other health worker that you have high blood pressure or hypertension*?*"* and if they said "yes" to question 1, then *(2) "Who told you"*? Hypertension diagnosis from qualified doctors (i.e., registered medically trained doctors or medical doctors with at least an MBBS degree) was defined if participants reported that they received the diagnosis from a qualified doctor in Question 2 [6].

#### Advice and treatment to control hypertension

Participants who reported yes to the question "Have ever been told by a doctor or other health worker that you have high blood pressure or hypertension?", were asked whether they received the following five pieces of advice to control blood pressure: medication, reduce salt, lose weight, smoking cessation, and increase exercise, *["Are you currently receiving any of the following treatment/ advice by a doctor or other health worker to control your blood pressure*?*"*: *(1) "Prescribed medication*?*"; (2) "Advice to reduce salt intake*?*; (3) Advice/treatment to lose weight*?*"; (4) "Advice/treatment to stop smoking*?*"; and (5) "Advice to start/do more exercise*?*"]* [6].

#### Potential factors

The following variables were selected to examine as potential factors associated with hypertension diagnosis from qualified doctors: age (in years), gender, education, wealth quintile, place of residence, and division of residence. Respondents’ self-reported age was categorized as ‘18–39’, ‘40–54’, and ‘55 or more’ years. Gender (i.e., female or male) was self-reported as well. We classified an individual as overweight or obese when the body mass index (BMI) was more than 25 kg/m2; the BMI was obtained by dividing body weight (in kg) by the square of height (in meter^2^) [26, 27]. The education level was grouped into ‘no formal education’, ‘primary (i.e., 1 to 5 school years)’, ‘secondary (i.e., 6 to 10 school years)’, and ‘college or above (i.e., 11 or more school years)’. The household wealth score was derived from principal component analysis of household items and construction materials; the score was then stratified into the following quintiles: poorest, poorer, middle, wealthier, and richest [6]. During the survey, people living in a municipal or city corporation were considered urban residents. During BDHS 2017–18, Bangladesh had eight divisions (i.e., the largest administrative unit of the country): Dhaka, Chattogram, Rajshahi, Khulna, Barisal, Sylhet, Rangpur, and Mymensingh [6].

### Statistical analysis

First, we described the overall characteristics of the respondents according to whether they were diagnosed with hypertension by a qualified doctor. For categorical variables, we used weighted percentages (%) and unweighted numbers (n) to describe the distribution and used chi-square tests to compare the distributions. We used mean and standard errors (SE) to describe continuous variables and compared the distributions with Student’s t-tests. Next, we reported the proportion (with 95% confidence intervals (CIs)) of people who were diagnosed by qualified doctors according to their sociodemographic/socioeconomic characteristics. The multicollinearity was tested with variance inflation factors using an artificial linear regression model. We also investigated the associated factors using simple and multivariable logistic regression. Lastly, we investigated the association between receiving hypertension-controlling treatment/advice and the diagnosis by qualified doctors. We reported the unadjusted odds ratio (UOR) and adjusted odds ratio (AOR) along with the 95% CI to report the associations. To obtain the estimates, we accounted for the hierarchical structure of the survey design (i.e., multistage cluster sampling) and sample weights. We used complete case analysis to handle missing data. The sample weights accounted for sample coverage, rural-urban and divisional population distribution, and non-response. The analyses were conducted with R (R Core Team, 2023).

### Ethical approval

The institutional review boards of the ICF International and Bangladesh Medical Research Council provided ethical approval for the BDHS 2017–18.

## Results

A total of 1,710 participants were included in the analysis; their mean age was 50.1 (SE: 0.43) years, and 68.3% were females (Table 1). Compared to participants who were not diagnosed by qualified doctors, those who received the diagnosis from a qualified doctor had a higher proportion of people with overweight/obesity (49.4% vs. 34.8%), college/above education (13.3% vs.7.6%), richest wealth quintile (40.2% vs. 19.5%), and urban residence (39.2% vs. 23.6%). On the other hand, people without the diagnosis from qualified doctors had a higher proportion of people with no formal education and the poorer/poorest wealth quintile. Approximately, a quarter (23.2%) of the respondents were from Dhaka division.

**Table 1:** Characteristics of the respondents according to diagnosis by qualified medical doctors^1^, Bangladesh 2017–18.

Variables		Self-reported diagnosed hypertension (n = 1710)	Diagnosis by qualified doctors, % (n)		
			Yes (n = 994)	No (n = 716)	p
**Age (in years)**	**Mean (SE)**	50.1 (0.43)	50.7 (0.54)	49.4 (0.64)	0.11
	**18–39**	26.3 (434)	23.4 (224)	29.8 (210)	0.024
	**40–54**	31.6 (543)	33.3 (328)	29.5 (215)	
	**55+**	42.2 (733)	43.3 (442)	40.8 (291)	
**Gender**	**Female**	68.3 (1170)	66.7 (672)	70.2 (498)	0.18
	**Male**	31.7 (540)	33.3 (322)	29.8 (218)	
**Overweight/obesity**	**No**	57.2 (984)	50.6 (509)	65.2 (475)	<0.001
	**Yes**	42.8 (726)	49.4 (485)	34.8 (241)	
**Education level**	**No formal education**	33.1 (538)	28.4 (267)	38.8 (271)	<0.001
	**Primary**	29.8 (526)	29.1 (290)	30.6 (236)	
	**Secondary**	26.4 (431)	29.2 (280)	23.0 (151)	
	**College/above**	10.7 (215)	13.3 (157)	7.6 (58)	
**Wealth quintile**	**Poorest**	12.5 (210)	9.5 (92)	16.1 (118)	<0.001
	**Poorer**	14.8 (249)	10.2 (101)	20.4 (148)	
	**Middle**	19.6 (319)	18.4 (164)	21.1 (155)	
	**Richer**	22.2 (368)	21.7 (210)	22.9 (158)	
	**Richest**	30.9 (564)	40.2 (427)	19.5 (137)	
**Place of residence**	**Rural**	67.9 (718)	60.8 (495)	76.4 (223)	<0.001
	**Urban**	32.1 (992)	39.2 (499)	23.6 (493)	
**Division of residence**	**Dhaka**	23.2 (223)	23.1 (128)	23.2 (95)	<0.001
	**Chittagong**	19.1 (247)	23.6 (169)	13.6 (78)	
	**Barishal**	7.0 (220)	7.9 (145)	5.8 (75)	
	**Khulna**	13.7 (259)	11.4 (131)	16.5 (128)	
	**Mymensingh**	6.9 (164)	6.2 (87)	7.8 (77)	
	**Rajshahi**	13.0 (194)	12.7 (111)	13.3 (83)	
	**Rangpur**	10.2 (188)	7.2 (86)	14.0 (102)	
	**Sylhet**	7.0 (215)	7.8 (137)	5.9 (78)	

1. Presented as weighted column percentages and unweighted numbers unless otherwise specified.

Table 2 describes the prevalence of diagnosis from a qualified doctor along with the factors associated with receiving the diagnosis. Overall, 54.9% (95% CI: 51.8% to 58.0%) of the people were diagnosed by qualified doctors. The prevalence and odds of the diagnosis were higher among older people than younger ones. People with higher education or household wealth had higher prevalence and odds than those with lower education or wealth. Urban residents (prevalence: 66.9%, 95% CI: 64.6% to 69.3%) had higher odds of diagnosis than their rural (prevalence: 49.2%, 95% CI: 47.2% to 51.2%) counterparts (AOR: 1.61, 95% CI: 1.18 to 2.21). We also found the association between divisions of residence and diagnosis.

**Table 2:** Prevalence and factors associated with diagnosis by qualified medical doctors among people with the diagnosis of hypertension, Bangladesh 2017–18.

Variable		Prevalence (95% CI) (n = 1710)	UOR (95% CI)	AOR (95% CI)^1^
**Overall**		54.9 (51.8,58.0)	--	--
**Age (in years)**	**18–39**	48.9 (46.1,51.7)	Ref.	Ref.
	**40–54**	57.9 (55.4,60.3)	1.44 (1.09,1.90)	1.60 (1.17,2.20)
	**55+**	56.4 (54.3,58.5)	1.35 (1.04,1.75)	1.85 (1.34,2.54)
**Gender**	**Female**	53.6 (51.8, 55.4)	Ref.	Ref.
	**Male**	57.6 (55.0,60.2)	1.18 (0.93,1.49)	1.03 (0.78,1.36)
**Overweight/obesity**	**No**	48.6 (46.7, 50.4)	Ref.	Ref.
	**Yes**	63.3 (61.1,65.5)	1.83 (1.46,2.28)	1.50 (1.17,1.94)
**Education level**	**No formal education**	47 (44.6,49.5)	Ref.	Ref.
	**Primary**	53.6 (51.0, 56.2)	1.30 (1.01, 1.69)	1.14 (0.86,1.53)
	**Secondary**	60.8 (58.1,63.5)	1.74 (1.31,2.32)	1.42 (0.99,2.02)
	**College/above**	68.2 (64.3,72.1)	2.42 (1.62,3.60)	1.71 (1.04,2.80)
**Wealth quintile**	**Poorest**	41.8 (37.7,46)	Ref.	Ref.
	**Poorer**	37.8 (34.5,41.2)	0.85 (0.55,1.29)	0.80 (0.52,1.23)
	**Middle**	51.5 (47.8,55.1)	1.47 (0.96,2.27)	1.31 (0.84,2.03)
	**Richer**	53.5 (50.4,56.6)	1.60 (1.05,2.43)	1.23 (0.79,1.92)
	**Richest**	71.5 (68.9,74.1)	3.49 (2.27,5.36)	2.19 (1.35,3.57)
**Place of residence**	**Rural**	49.2 (47.2,51.2)	Ref.	Ref.
	**Urban**	66.9 (64.6,69.3)	2.09 (1.61,2.71)	1.61 (1.18,2.21)
**Division**	**Dhaka**	54.9 (51.1,58.6)	Ref.	Ref.
	**Chittagong**	67.9 (64.0,71.8)	1.74 (1.10,2.75)	2.18 (1.34,3.54)
	**Barishal**	62.5 (58.3,66.7)	1.37 (0.87,2.17)	2.29 (1.37,3.82)
	**Khulna**	45.7 (42.1,49.4)	0.69 (0.46,1.05)	0.98 (0.61,1.56)
	**Mymensingh**	49.2 (44.2,54.3)	0.80 (0.49,1.31)	1.34 (0.78,2.31)
	**Rajshahi**	53.7 (49.3,58.1)	0.95 (0.60,1.50)	1.60 (0.95,2.69)
	**Rangpur**	38.5 (34.0,42.9)	0.51 (0.32,0.83)	0.84 (0.50,1.41)
	**Sylhet**	61.7 (57.0,66.4)	1.32 (0.81,2.16)	2.21 (1.31,3.74)

Abbreviations: UOR: Unadjusted odds ratio; AOR: Adjusted odds ratio; CI: Confidence interval

1. Adjusted for all variables in the column.

When we investigated the association between receiving hypertension-controlling advice/treatment and diagnosis by qualified doctors, we observed that the prevalence and odds of receiving any advice were significantly higher among people who were diagnosed by qualified doctors compared to those who were not diagnosed (Table 3). For instance, the overall prevalence of receiving recommendations about reducing salt intake was 66.5% (95% CI: 63.3% to 69.7%); it was 76.6 (95% CI: 73.2% to 80.0%) among people who were diagnosed by qualified doctors and 54.2% (95% CI: 49.4% to 59.0%) among people who were not diagnosed by qualified doctors. The AOR for receiving that information was 2.36 (95% CI: 1.80 to 3.10).

**Table 3:** Association of diagnosis by qualified medical doctors with receiving hypertension controlling advice and treatment, Bangladesh 2017–18.

Advice	Diagnosis by qualified doctors, % (95% CI)^1^			UOR (95% CI) (Ref.: No)	AOR (95% CI) (Ref.: No)
	Overall (n = 1710)	Yes (n = 994)	No (n = 716)		
**Drug**	79.5 (76.5,82.5) (n = 1373)	84.2 (81.3,87.0) (n = 845)	73.8 (69.2,78.5) (n = 528)	1.88 (1.43,2.48)	1.73 (1.27,2.36)
**Reduce salt**	66.5 (63.3,69.7) (n = 1144)	76.6 (73.2,80.0) (n = 762)	54.2 (49.4,59) (n = 382)	2.94 (2.28,3.79)	2.36 (1.80,3.10)
**Lose weight**	35.8 (32.6,39.0) (n = 620)	46.3 (42.1,50.5) (n = 462)	22.9 (18.9,26.9) (n = 158)	2.76 (2.15,3.55)	2.58 (1.97,3.37)
**Stop smoking**	32.3 (28.9,35.6) (n = 577)	40.1 (35.7,44.5) (n = 405)	22.7 (18.7,26.7) (n = 172)	2.28 (1.74,2.97)	2.22 (1.66,2.96)
**Exercise more**	42.6 (39.3,45.9) (n = 732)	54.1 (49.8,58.4) (n = 535)	28.7 (24.5,32.8) (n = 197)	2.90 (2.22,3.79)	2.34 (1.77,3.09)

1. Presented as column percentage with 95% CI.

Abbreviations: UOR: Unadjusted odds ratio; ratio; AOR: Adjusted odds ratio; CI: Confidence interval

* Adjusted odds ratios were obtained from logistic regression with adjustment for age, gender, overweight/obesity, education, wealth quintile, place of residence, and division

In S1 Table, we also investigated the odds of receiving advice and treatment among people with overweight/obesity and observed that a substantial proportion of them did not receive adequate advice, including weight loss advice.

## Discussion

In this study, only slightly more than half of the respondents with known hypertension received their diagnoses from qualified doctors. Then, compared to people with lower SES and rural residence, those with higher SES (i.e., increased wealth and education) and urban residence had higher prevalence and odds of receiving the diagnosis from qualified doctors. Additionally, we observed that those diagnosed by qualified doctors had higher odds of receiving hypertension-controlling advice and treatment than those who were not. This study adds to the growing body of literature regarding hypertension care in an LMIC like Bangladesh.

Although we investigated the impact of receiving a hypertension diagnosis from qualified doctors on providing hypertension-controlling advice and treatment, it is essential to note that some people may not require all the advice or treatment. For instance, ’weight loss’ advice is only applicable to overweight/obese people [11]. They may also require other measures like smoking cessation (i.e., if they are smokers), salt reduction (i.e., if they consume extra salt), or exercise promotion (i.e., if they don’t have sufficient physical activity). Therefore, we additionally investigated the odds of receiving advice among people who are overweight/obese and found that a substantial proportion of them did not receive weight loss advice (S1 Table). The prevalence and odds of other advice were also lower among the people who were not diagnosed by qualified doctors. Our findings underscore the importance of receiving advice and treatment from qualified doctors.

Due to the lack of previous literature on hypertension care in Bangladesh, we were not able to compare our findings to prior articles; however, as we mentioned earlier, our observed socioeconomic and rural-urban disparities were reported for other types of care as well [6, 12–15]. While individuals with low SES may be at a lower risk of hypertension, they may experience more significant negative consequences due to inadequate utilization of appropriate care [17, 28]. It is essential to consider the reasons for these disparities. Socioeconomically disadvantaged people may have less access to healthcare due to affordability issues. Rural people may have lower access due to fewer qualified doctors in rural regions than in urban areas [16–18]. We also observed that the younger people had lower odds of receiving advice and treatment than older people. The people also had lower odds of hypertension diagnosis or control [17, 28]. Reducing these disparities and expanding coverage of care provided by qualified doctors is crucial in alleviating the burden of hypertension and its complications. Conducting population-based surveys to investigate the reasons and barriers to the underutilization of treatment by qualified doctors is essential.

People with hypertension have a higher likelihood of many other NCDs (e.g., diabetes, dyslipidemia, and metabolic syndrome), and they share many common risk factors (e.g., overweight/obesity and physical inactivity) [29–31]. Therefore, following the hypertension control advice and treatment would not only control it or minimize its future complications but also reduce the burden of other similar health conditions. Managing hypertension is essential for its direct effects and broader positive impacts on overall health and the healthcare system. Overall, adhering to appropriate treatment and control measures can significantly impact the health system and reduce the NCD burden in the country. In Bangladesh, people can receive diagnosis and treatment from qualified doctors at governmental health centers for a minimal fee [6]. People can also receive treatment from private healthcare providers [6, 7]. The government has established NCD corners in upazila (i.e., subdistrict) health complexes. Previously published reports demonstrated the lack of resources (e.g., screening instruments, antihypertensive drugs, and tobacco cessation counseling services) in these centers; therefore, despite having a minimal fee, patients may end up paying a large amount of extra money for the diagnosis and treatment [25, 32]. Without increasing the health centers’ capacity, ensuring adequate healthcare coverage would be challenging.

The "Multisectoral Action Plan for Prevention and Control of NCD 2018–2025" is in place to tackle the burden of NCDs in Bangladesh. The execution of the action plan utilizes a ’health in all policies’ strategy, involving stakeholders beyond the health sector to address and shape public policies related to common risk factors, promoting a holistic approach to health across various sectors. Successful implementation of the action plan would be critical to controlling hypertension and other NCDs [33, 34]. Adequate monitoring is essential, given that most previous government-initiated NCD programs in Bangladesh were unsuccessful due to inadequate planning, implementation, management, or monitoring [33]. While the plan includes coordination between different levels of health centers and providing training to health workers, it is worth noting that successful examples from child health, such as the World Health Organization’s Integrated Management of Childhood Illness (IMCI) program, can be instructive [35]. The IMCI was crucial in improving child healthcare in Bangladesh [36]. The program trains primary healthcare workers on common childhood diseases (e.g., pneumonia and diarrhea) [35]. The government can adopt similar programs to train informal healthcare providers to tackle hypertension and other common NCDs (e.g., diabetes and dyslipidemia) [35, 37]. In addition to addressing the lack of training, several other barriers to effective treatment could be removed, including supervision, monitoring, coordination, and guidelines in the local language (i.e., Bengali) [22, 38]. Community-based programs have shown effectiveness as well [39].

Our study possesses several notable strengths. First, we used a nationally representative sample that covered rural and urban regions of all administrative divisions and had a large sample size. The survey used a standardized and validated questionnaire to collect data. However, the limitations of our study also warrant discussion. The data were based on self-reports; therefore, there were chances of recall errors. Some people may also misidentify the qualified doctors. This was a cross-sectional study, and the lack of temporal certainty about cause-effect relationships was another issue; for instance, the survey asked that if the participants were ever diagnosed with hypertension, some people could relocate from rural to urban (and vice versa) following the diagnosis. Apart from overweight/obesity, we did not account for other comorbidities (e.g., diabetes and dyslipidemia), which may increase the likelihood of diagnosis by qualified doctors. We also relied on self-reports about hypertension instead of actual measurement or verifying medical records; therefore, some misclassification may occur, and future studies should consider actual measurement or medical records.

## Conclusion

This study addressed socioeconomic and rural-urban disparities regarding hypertension diagnosis and care from qualified doctors in Bangladesh. Socioeconomically disadvantaged and rural residents may suffer more from the negative consequences of hypertension. Reducing these inequalities would be challenging; however, it would be crucial to cure the country’s hypertension burden. Although the government has taken initiatives, more efforts, coordination, and training are required to increase their success.

## Supporting information

S1 Table Prevalence and association of diagnosis by qualified medical doctors with hypertension-controlling advice and treatment among overweight/obese people. (DOCX)
