# Supplementary material for: Disparities and factors affecting hypertension diagnosis from qualified doctors in Bangladesh and its impact on receiving hypertension control advice: Analysis of demographic & health survey 2017–18
Source: PLOS Glob Public Health. 2024 Jul 23;4(7):e0003496. doi: 10.1371/journal.pgph.0003496 (PMC11265666; doi:10.1371/journal.pgph.0003496)
Supplement: S1 Table — (DOCX) [file pgph.0003496.s001.docx]

**S1 Table: Prevalence and association of diagnosis by qualified medical doctors with hypertension-controlling advice and treatment among overweight/obese people**

| **Advised** | **Overall** | **Yes** | **No** | **UOR** | **AOR** |
| --- | --- | --- | --- | --- | --- |
| Drug | 81.1 (77.6,84.5) | 85.0 (81.4,88.6) | 74.3 (67.6,81.0) | 1.96 (1.26,3.05) | 1.63 (0.98,2.71) |
| Reduce salt | 70.2 (66.1,74.3) | 78.2 (74.0,82.4) | 56.2 (49.1,63.3) | 3.33 (2.21,5.01) | 2.71 (1.78,4.12) |
| Lose weight | 49.6 (44.9,54.3) | 59.5 (54.0,65.1) | 32.5 (25.7,39.4) | 2.79 (1.94,4.02) | 2.57 (1.73,3.81) |
| Stop smoking | 31.1 (26.3,35.9) | 38.1 (31.9,44.3) | 19.0 (13.3,24.6) | 2.63 (1.7,4.09) | 2.38 (1.46,3.88) |
| Exercise more | 53.0 (48.2,57.8) | 63.7 (58.1,69.3) | 34.5 (27.0, 42.0) | 3.05 (2.08,4.48) | 2.65 (1.77,3.97) |
